# Patient and Healthcare Professionals Perspectives on the Delivery of Exercise Education for Patients With Type 1 Diabetes

**DOI:** 10.3389/fendo.2019.00076

**Published:** 2019-02-19

**Authors:** Ian Litchfield, Rob C. Andrews, Parth Narendran, Sheila Greenfield

**Affiliations:** ^1^Institute of Applied Health Research, University of Birmingham, Birmingham, United Kingdom; ^2^Medical School, University of Exeter, Exeter, United Kingdom; ^3^Taunton and Somerset NHS Foundation Trust, Musgrove Park Hospital, Taunton, United Kingdom; ^4^Institute of Immunology and Immunotherapy, University of Birmingham, Birmingham, United Kingdom; ^5^University Hospitals Birmingham NHS Foundation Trust, Queen Elizabeth Hospital, Birmingham, United Kingdom

**Keywords:** type 1 diabetes (T1D), exercise, service delivery and organization, structured education program, staff development

## Abstract

**Objective:** One way of improving the prognosis for the growing numbers of people with type 1 diabetes (T1D) is to increase their frequency of exercise. One known barrier to this is the lack of cohesive support and information from care providers. To better understand the issues around existing support for patients wishing to exercise and inform the design of an education package specifically to facilitate safe exercise we interviewed care providers and patients about the existing provision of support.

**Research Design and Methods:** The study was based within two large UK teaching hospitals where four focus groups were undertaken two consisting of patients diagnosed with T1D who undertook regular exercise, and two with health care providers (HCPs) that were part of the diabetes care team. In all 14 patients and 11 staff were involved. These were complemented by two 1:1 interviews with staff unable to attend group discussions.

**Results:** We found the successful provision of education and advice was influenced by factors relating to the individual patient and their service provider. Patient factors included the type of activity and complexity of the exercise regime, the level of engagement with their condition and care and health literacy. Service-related factors included inconsistent training, a lack of capacity and continuity, and limited coherence of information from across their care team.

**Conclusions:** Any education package developed to support exercise in patients with type 1 diabetes should be offered at a time following diagnosis in accordance with patients' preferences and priorities, contain information on how to manage regular and irregular bouts of exercise. Patients described how they related more closely to the stories of their peers than famous sports stars and one way this can be facilitated is by group delivery. The content and relevance of any supporting materials should be closely considered. Training in the delivery of a novel education package should be made available to staff across the care team to enable them to either deliver the course or increase their confidence in offering salient advice as part of routine care.

## Introduction

The number of individuals affected by Type 1 Diabetes (T1D) continues to increase with some 20 million affected globally (1). In the United States the estimated 3 million with the condition is set to treble by 2050 (2, 3) with a concomitant increase in economic burden that is currently estimated at some $14.4 billion annually (4). One way in which the effects of T1D might be mitigated and the associated costs might be reduced is by meeting the recommendations for patients with T1D to undertake regular moderate-intensity exercise (5–9)^1^. The potential benefits both for patients and service utilization of their maintaining such a regime are numerous (10, 11) including a reduced risk of cardiovascular disease (12, 13), microvascular complications, hypoglycaemia (14) as well as enhanced psychological well-being (15). Existing guidelines recommend that patients with T1D should exercise regularly^1^,^2^ however, with a reported 50% undertaking little or no physical activity either in Europe (14) or the United States (16), the need to increase the amount of sustained exercise and physical activity undertaken by patients with T1D remains^1^(17). To this end research on both sides of the Atlantic (18–21) has identified a number of barriers that might inhibit patients with T1D exercising including a lack of concerted information on exercise management. In the United States, it's recommended that patients seek the advice of physiologists or fitness coaches with experience of managing patients with T1D (22) whereas in the UK its expected support will be provided by dieticians, specialist diabetic nurses, educators and consultants as part of their routine care^1^.

The content of any advice is frequently generic focussing on the basics of carbohydrate counting and insulin management. However, the implementation of this advice is influenced by numerous factors such as the duration and intensity of physical activity (23), the presence of co-morbidities (24), the level of patient engagement (25), and environmental parameters such as temperature (26). All of these contribute to variable glycaemic responses that make uniform recommendations challenging (22). In the UK patients attempting to negotiate these multiple influences are often dependent upon multiple care providers, with diverse levels of training and experience (27–29) with a recent survey reporting that two thirds were unfamiliar with the evidence-based guidance^1^ leaving them unable to offer basic advice on insulin action (27).

There appears to be a clear need in T1D care both in the UK and elsewhere for a structured educational package specifically addressing safe exercise. Our study “Supporting adults with Type 1 Diabetes to undertake exercise; Developing and piloting an Education programme for exercise in Type 1 Diabetes” (EXTOD) was established to co-design an education package to support the management of exercise by both patients and staff^3^. The initial phase used focus groups to better understand the existing provision of advice and education on exercise that explored both patient and provider perspectives and preferences. The findings presented here describe for the first time the existing influences on how successfully the content of the information provided and the processes by which it is delivered meets the needs of patients. In doing so we offer valuable insight into the requirements of patients with T1D and their care providers as they attempt to maintain or increase levels of exercise. Our findings can inform the actions of all providers supporting patients with exercise and have been integral in the development of the EXTOD educational package.

## Methods

### Settings

The study took place in two hospitals in the UK. Taunton and Somerset NHS Foundation trust which is a medium sized hospital in the Southwest that provides care for people living in a mixture of rural and urban environments and the Queen Elizabeth Hospital Birmingham. This is a large teaching hospital in the West Midlands that provides care for people living predominantly in an urbanized Metropolitan environment.

### Recruitment

#### Patients

To participate in the study patients must have a clinical diagnosis of T1D, be aged between 18 and 70, be on a basal bolus insulin regime, and have attended their local approved T1D Education Programme [DAFNE, BERTIE, Living with diabetes, or equivalent^4^,^5^ (30)]. Finally they should be exercising regularly or starting to train for a specific sporting event. All eligible patients were approached directly in diabetes clinics in a consecutive manner in each hospital by healthcare professionals aware of their existing or planned exercise activities. Those that expressed an interest were provided with an information sheet and offered an appointment with the research nurse who would answer their queries, discuss eligibility, and consent participants.

#### Staff

Any Health Care Professional (HCP) involved in the care of patients with T1D across the Southwest and West Midlands were contacted via email by the research teams in each hospital to ask if they would be interested in taking part in this study. As with patients those that expressed an interest were provided with an information sheet and offered an appointment to see the research nurse to discuss their involvement, to check their eligibility and to be consented.

#### Ethics

This study was carried out in accordance with the recommendations of the National Health Service Health Research Authority, through the Coventry and Warwickshire research Ethics Committee. The protocol was approved by the same committee and given the reference code 16/WM/0034. All subjects gave written informed consent in accordance with the Declaration of Helsinki.

#### Data Collection

We collected data on experiences of existing exercise education provision from staff and patients using focus groups or for those unable to attend the groups by semi-structured telephone interviews. Focus groups were chosen as the primary method of data collection as they offer opportunity for participants to reflect and test ideas rather than formulate ideas on the spot (31). The uninhibited discussion can also serve to remind participants of their past experiences and produce new thoughts about the delivery of education on exercise. In this study, the ability to generate information on collective views is useful to generate consensus to inform the nascent education package (31).

Two focus groups, were convened at each hospital (32). One was comprised of people with T1D and the second multi-disciplinary HCPs involved in T1D patient care. Each group was facilitated by IL a research fellow with extensive experience of qualitative methodologies used in improving health service delivery. A topic guide was used, tailored to either patients (see Box 1) or providers (see Box 2) and designed to allow themes and issues to arise naturally from the interaction of the group during the course of the meeting (33).

**Box 1:** Patient Topic guide

*Your own exercise programme*	
1. Can you tell me about the sort of exercise you regularly take each week?	
*** Prompts;***	When, who with, length/intensity/frequency, type of sport
2. Do you do anything special to prepare yourself – before, during or after exercising?	
*** Prompts:***	Food, diet, drinks - including alcohol
3. Do you have any problems when you exercise?	
*** Prompts:***	Tiredness, muscle fatigue, other
4. Is there anything that makes it easier/more difficult to exercise?	
*** Prompts:***	T1D, other medical condition, related non-medical factors
*Current provision of information around exercise and managing your condition*	
5. Who have you spoken with about T1D and exercise?	
*** Prompts:***	Consultant, other hospital doctor, GP, nurse, dietician, other
6. When did you discuss this?	
*** Prompts:***	At diagnosis, at each consultation, every so often, a specific session
7. What was the content of the discussion?	
** Prompts:**	Condition related - insulin, glucose levels, what to do about hypos Other health related - exercising when feeling unwell (colds etc.) Exercise related - different types of exercise and how much Preparation for exercise – steps to take during and recovering from exercise
	Diet - different foods, liquid and alcohol intake Environmental factors - the influence of hot/cold weather Personal characteristics – age, any other barriers and facilitators
8. Did you use additional sources of information?	
** Prompts:**	Webinar, peer group, online, correspondence course, booklet

**Box 2:** Staff topic guide

*Current provision of information around exercise and T1D*	
1. What aspects of exercise do you usually discuss with patients?	
** Prompts:**	The impact of different sports, length/intensity/frequency aerobic vs. anaerobic
2. When do you introduce this topic	
** Prompts:**	At diagnosis, at each consultation, every so often, in a separate or targeted session (e.g. DAFNE)
3. Are there any particular types of patients with whom you find it easier/more difficult to talk about exercise with?	
** Prompts:**	Age, ethnicity, gender, preferred exercise
4. What are the barriers if any to your discussing exercise?	
** Prompts:**	Time, lack of knowledge
5. Which healthcare professionals should talk to patients about exercise?	
** Prompts:**	Consultant, other hospital doctor, GP, nurse, dietician, other
6. What sorts of issues do you discuss?	
** Prompts:**	Condition related - insulin, glucose levels, what to do about hypos; Other health related - exercising when feeling unwell (colds etc.) Exercise related - different types of exercise and how much, Preparation for exercise – including steps taken during and recovering Diet - different foods, liquid and alcohol intake Environmental factors - the influence of hot/cold weather

#### Analysis

Focus groups in the South West were conducted and analyzed first. These began with the patient focus group and were followed by the HCP group. This allowed for a preliminary analysis to inform the topic guide of the second set of focus groups conducted in the West Midlands. Staggering the focus groups and their analysis helped ensure that the key points were recognized and explored. Semi-structured interviews were carried out with participants unable to attend by the researcher using the same topic guide as in the focus groups.

The patient and staff transcripts from the focus groups conducted in the South West were independently coded by IL and SG a medical sociologist. Although IL moderated the focus groups and conducted the interviews the potential for bias was checked by the close involvement of SG who was not involved in data collection. All data were analyzed using thematic analysis (33) and underwent three key phases of coding. The first involved multiple detailed codes relating to the various responses of the participants following the first two focus groups. During the second phase these were grouped into broader over-arching themes which IL discussed with SG who had independently read the same two transcripts. The final phase was again conducted with SG where the over-arching themes and sub-themes were agreed following the final two focus groups and 1:1 interviews. IL and SG then met and agreed emerging themes and sub-themes from across both patient and staff groups. This then became the coding framework for the remaining focus groups at the West Midlands site and the semi-structured interviews. Initially the patient and HCP transcripts were coded separately but when IL and SG discussed the coding it was apparent that the initial overarching themes extended across all the interviews. As a result the data from all transcripts were combined and the results presented together.

Group discussions were digitally audio-recorded with permission, and transcribed verbatim for analysis, Data were managed using N-Vivo software. The intention in this instance was not to reach data saturation but instead to use the content of the various discussions to inform the content and format of a novel education package^3^. However, by the end of the four focus groups and interviews no new themes or sub-themes were emerging.

## Results

### Patients

Two focus groups were conducted consisting of a total of 14 patients. They were aged from 22 to 71 of which eight were males and six female. The group in the South West (SW) contained four male and four female participants and the time since diagnosis varied from 3 to 50 years. Those that exercised or competed regularly were predominantly found in the SW group, where five of the eight regularly exercised a minimum of four times a week. The discussion here lasted 80 min. In the West Midlands (WM) group there were four male participants and two female with a slightly broader range in the time since diagnosis than the SW group of between two and 60 years. They tended to exercise less in this group with only one participant undertaking exercise more than three times a week. The discussion lasted 86 min. The characteristics of all patient participants are summarized in Table 1.

**Table 1:** Patient participants.

**Study ID**	**Time since diagnosis (years)**	**Exercise regime (Sessions per week)**
**SOUTH WEST (SW)**		
P1	3.5	Gym x2, Karate x2
P2	20	Dance x4, Perform x1
P3	5	Hockey training x4, Play at the weekend
P4	3	Football x1, Cycle commute and run regularly
P5	50+	Gym x2, Keep fit class x1
P6	5.5	Horse riding x7 (Competes on weekends)
P7	3	Gym x3
P8	4	Cycling x2, Gym x1
**WEST MIDLANDS (WM)**		
P9	17.5	Gardening, Aikido x3
P10	2	Swim x1, Keep fit x1
P11	56	Gardening, Walking, Infrequent cycling
P12	36	Walking short distances x7
P13	6	Badminton x3
P14	6	Squash x1, Cycling x5

### Health Care Professionals

A total of 11 staff were interviewed in the two focus groups; of the five participants in SW three were dieticians and two were specialist diabetes nurses with between 6 and 21 years experience, four of them had taught on DAFNE or equivalent courses. The discussion lasted 69 min. The group from the West Midlands consisted of three specialist nurses, two dieticians and a speciality doctor, their experience ranged from between 4 and 15 years, four had experience of teaching on DAFNE or equivalent courses and the group ran for 75 min. Two semi-structured interviews were conducted both with consultant diabetologists from the South West (HCP6 and HCP7) who were unable to attend the focus groups as originally intended. The interviews lasted between 45 and 55 min. The characteristics of staff participants are summarized in Table 2.

**Table 2:** Staff participants.

**Study ID**	**Job title**	**Time in post (years)**	**Exercise education provided**
**SOUTH WEST**			
HCP1	Diabetes dietician	21	Teach on carbohydrate counting course (plus 4 h a week research)
HCP2	Diabetes dietician	8	Teaches on carbohydrate counting course, specialist interest in sport
HCP3	Diabetes dietician		Pediatrician (previously adult) Teaches on carbohydrate counting course
HCP4	Diabetes specialist nurse	6	Teaches on carbohydrate counting course
HCP5	Diabetes specialist nurse	12	1 day a week “pumps adviser”
HCP 6	Consultant in diabetes endocrinology, acute and general internal medicine	13	Proactive enquiry around exercise during consultation. Advises on range of exercise and activity
HCP 7	Consultant diabetes endocrinologist, and the clinical lead for patient specialities	8	Advises patients on recreational exercise and events such as marathons
**WEST MIDLANDS**			
HCP8	Speciality Doctor	8	Educates patients on exercise in response to their enquiries
HCP9	Diabetes dietician	2	Deliver the exercise section of the carbohydrate counting course.
HCP10	Specialist dietician	4	Teaches exercise as part of education programmes for both Type 1 and Type 2 diabetes.
HCP11	Diabetes specialist nurse	15	Teaches on carbohydrate counting course
HCP12	Diabetes specialist nurse	15	Teaches on carbohydrate counting course
HCP13	Diabetes specialist nurse	14.5	Teaches on carbohydrate counting course

### Analysis

Themes were combined across patient and staff groups and four emerged which are presented together below. The first concerns the nature of the exercise regime undertaken by each individual relating to both its complexity in terms of the types and combination of exercise and the consistency in the pattern of exercise. The second theme centers on patient engagement, specifically how their ability to manage health is affected by the knowledge, skills and confidence they possess as reflected in their preferences, the use of self-management techniques and level of health literacy. The third theme is related to organizational factors concerning staff training, limits of capacity, consistency of care, and existing education strategies. These themes and sub-themes are summarized in Table 3 and below we present exemplar quotes for each alongside the key characteristics of the participant that provided them. Additional and extended quotes can be found in Supplementary Table 1.

**Table 3:** Influences on the successful delivery of exercise education: themes and sub-themes.

**Theme**	**Sub theme 1**	**Sub theme 2**
1.0 Exercise regime	1.1 Type of exercise	Predictable intensity Anaerobic exercise Specialized sport
	1.2 Patterns of exercise	Routine vs. sporadic
	1.3 Intensity	Competitive vs. non-competitive
2.0 Patient engagement	2.1 Patient preference	Priority of exercise
	2.2 Self-management	Monitoring Carbohydrate counting
	2.3 Health Literacy	Understanding advice
3.0 Organizational factors	3.1 Staff training	Limited knowledge of the effects of exercise Deskilling
	3.2 Capacity	Limited access to education packages Limited access to specialist providers Length of consultation
	3.3 Coherence of care	Consistency of message Continuity of care
4.0 Existing education strategies	4.1 Structured education package	Criteria for inclusion
	4.2 Format and content	Patient stories Generic written information

### Exercise Regime

The types of exercise individuals enjoyed, the consistency of their exercise regime and the intensity of the activity all impacted on the ability of staff to advise them how to safely conduct this exercise.

### Type of Exercise

#### Predictable Intensity

The nature of the exercise being undertaken was a key indicator of how confident staff felt in delivering appropriate advice. Staff described feeling most comfortable presenting advice to individuals who took part in relatively consistent bouts of aerobic exercise such as long-distance running or cycling.

“… the longer distance runners, triathletes, and cyclists I tend to find a bit easier to work with, …maybe because their activity is a bit more predictable? They tend to have…more structured training, and more obvious responses to their activity.”

- *HCP6 Consultant*

Patients reflected on how difficult it was to accurately predict the varying intensity of an individual bout of exercise and the difficulty this presented in managing their body's response.

“…with the intensity, because that varies depending on just even how you're feeling on the day… you might not feel like you want to go too far…and therefore you get different readings than what you might have done a week ago doing the same distance, or length in time of exercise, or whatever.”

–* Patient 8*

#### Anaerobic Exercise

A notable area of uncertainty was instructing patients about anaerobic exercise such as weight-training. One diabetic specialist nurse (DSN) described how it felt counter-intuitive that blood sugar levels should rise after lifting weights and the struggle they had in coming to terms with the concept.

“I still find it a bit hard around the anaerobic sport, when they're doing weights and things like that, and actually their blood sugars go up, so one guy I mentioned he does that first thing in the morning, so he has no breakfast and three units of insulin and goes to the gym, and then his blood sugars are okay. I sort in my head think “Oh!” [laughter] but it does work. But that took a lot on both of us gradually edging that up, but it doesn't seem right does it?”

- *HCP5 Diabetes Specialist Nurse*

Patients also recognized the different effects on their physiology of aerobic as opposed to anaerobic exercise.

“ …you know what to do for let's say, I don't know, spin class, you know how you need to fuel yourself for that, then if you want to change what exercise you do, if you want, I don't know… a spin class and a weight class affects me completely differently and I have to fuel differently for that,”

–* Patient 7*

#### Specialized Sport

Staff spoke about how their personal experience of a particular sport would inform the advice they provided. Where patients undertook more specialized or unusual forms of exercise they felt less able to offer advice. For example one DSN described how her lack of understanding of the physical demand of extreme triathlons made it harder for her to provide relevant advice.

“I get quite… we've got a couple that one is training to do the iron man competition, and one is doing a triathlon and I have absolutely no idea where to start, because I don't understand the sports themselves and exactly what they're actually trying to achieve. So not knowing what they're doing it's hard to give advice when you don't know, and I'm certainly not going to go and do a triathlon [laughs].”

- *HCP13, Diabetes Specialist Nurse*

### Patterns of Exercise

#### Routine vs. Sporadic

Whether the individual's exercise regime was predictable impacted on how readily it might be managed and the level of sophistication of advice and education they required. For example one patient expressed their uncertainty of how to manage change in their exercise routine.

“…it's any kind change to your routine, if you can stick to your routine you're fine, but I find any kind of change is when I have problems.”

-Patient 10

Staff also described the issues they had informing patients who took on a variety of sports in various combinations. For example, one dietician described their apprehension in advising those that played an aerobically intense sport like rugby in the winter then a sport of lengthier duration with bursts of aerobic and anaerobic exercise such as cricket.

“I think people that have a routine where they do things, the same exercise, are easier to advise, but those people like you were saying who do a different sport, or do cricket in the summer, rugby in the winter, and actually need to do very different things, and there's parts of the year when you're doing both.”

– *HCP5, Diabetes Specialist Nurse*

### Intensity

#### Competitive vs. Non-competitive

Another factor central to the level of confidence of staff in speaking to patients about managing exercise was the standard at which individuals exercised or played sport. In the view of one dietician the sporting ability of a patient would impact on their own confidence to offer appropriate advice.

“I think my difficulty comes when I've got people who are very sporty, either competitive sport or are training for something specific like for a marathon or something. Then I feel it stops my confidence not theirs, and that might rub off on them, I don't know, because then it becomes quite difficult, because you're managing quite a lot regarding their diabetes, and I suppose that's just experience of what to do with people that are very sporty or training…”

– *HCP12, Diabetes Specialist Nurse*

One patient described how the impact of aerobically demanding competitive sport is more difficult to manage.

“I used to event, luckily I don't event anymore, I just do dressage, so it's very controlled, so it's not a huge high impact thing. You work hard but your heart rate never [rapidly increases]… it probably does through adrenaline, but not like a run or if you're cycling hundreds of miles, it's quite controlled.”

–* Patient 6*

### Patient Engagement

Patient engagement is defined as an individual's knowledge, skills, ability, and willingness to manage their own health and care^6^. Here we describe three factors related to the level of this engagement amongst our patients and how it shaped the support they requested, received, and assimilated. These factors are, their preference for the content of consultations, the level of competence in their self-management, and related to this, their level of health literacy.

### Patient Preference

#### Priority of Exercise

We found that the content of any advice provided around exercise was frequently led by patient enquiry and related to their preferences and priorities. If regular exercise was a central activity prior to diagnosis then patients would be more likely to ask additional questions and require greater detail in the response. In this example a DSN described how the importance of exercise to the individual would be apparent from the first consultation and shape subsequent conversations.

“It all depends very much on the patient because you might have somebody who is newly diagnosed who is a regular sportsperson and they're doing a particular activity, and they want to know whether they can keep it going, and they would introduce the fact that they need to have the advice, whereas you might have another person who is fairly newly diagnosed who actually wants to just concentrate on getting to terms with diabetes without looking at other things that can affect it. So it's all very much down to the individual.”

– *HCP13, Diabetes Specialist Nurse*

### Self-Management

Self-management describes the capability of an individual to recognize, treat, and manage their own health whether independently or in partnership with the healthcare system^7^. The basic advice offered to any patients with T1D irrespective of exercise regimen involves a number of self-management techniques, in particular how to monitor and record blood glucose levels, and count the carbohydrates they have consumed. The ability or willingness of patients to consistently fulfill these tasks would vary.

#### Monitoring

For clinicians to provide salient advice they must have some understanding of an individual's metabolic response to the exercise they undertake and the carbohydrates they consume. One patient who cycled for lengthy periods described how they would monitor their blood glucose sugar as often as every 30 min.

“…you've got to try and work it out like we've said before. But I think they said that every half an hour you've got to re-evaluate the situation, so when you're doing something like four and a half hours, I'm sure with cycling and things, if you do cycling you've got to look at it every half an hour and just take on fuel, it's hard work.”

- Patient 4

However, some patients outwardly interested in undertaking regular exercise would fail to provide any information on their blood glucose levels. In its absence providers felt unable to offer relevant advice. For example a dietician described an instance where a patient wanted to increase their level of exercise without collecting or presenting the relevant information.

“…and I think the problem is we are generally presented with a person who maybe wants to increase activity or who has got problems, but presents you with no testing and no information, and then you say to them “Well you've got to go away, you've got to do some testing….”

– *HCP5, Diabetes Specialist Nurse*

#### Carbohydrate Counting

A basic knowledge of carbohydrate counting is necessary to exercise safely with T1D yet patients did not always possess this capability. One dietician described how patients with a keen interest in sport would not be able to complete the basic task of carbohydrate counting.

“Some people who come to you want to specifically talk about their interest in sport and how to manage it. But I find that some of them aren't even carbohydrate counting or have any of the foundations. So where you've set out to think this is all going to be targeted around sport you then begin stripping it back and doing all the foundations of the basic principles or carbohydrate counting and dose adjustment. So I found that it's pointless giving much information until they've even got that foundation, because otherwise that's more important than then tailoring for all the sports advice.”

– *HCP2, Diabetes Dietician*

Related to this a patient described how they only learnt to control their body's response to training after an extended period of trial and error.

“Mine is a little bit involved with diet as well. So I tend to reduce the amount of insulin I'm having if I'm training, but if I'm performing at the weekend? I tend to carb load the day before, and then I have less [food] but fully protein based the day off, so that [way] my liver doesn't go into overdrive with my blood sugar spikes. It took me quite a while to work out how to manage that.”

–* Patient 2*

### Health Literacy

#### Understanding Advice

The term health literacy describes the ability of a patient to understand and assimilate health-related information^8^. Health literate patients with T1DM were able to independently use the written information contained within the booklets or pamphlets provided as part of their structured education package. For example a patient described how one such booklet provided by their diabetes team remained a useful reference point and allowed them to cope with some of the less common side-effects.

“I know that when I've been quite ill and I've used sick day rules through DAFNE, I'm glad I can grab that leaflet and that booklet and I can read, because it's hard to remember it because you don't often get ketones when you get ill, but if you've got good control you know you're getting ill and you can keep your levels low enough that you don't get ketones.”

– *Patient 6*

In terms of communicating to patients one HCP described how they would use the “talking test” to help a patient understand the various levels of intensity of their exercise.

Talk about the talking test, whether they can talk and how red in the face they go, that's what we talk about, to calibrate it.

- HCP7

### Organizational Factors

Organizational factors are related to the levels of staff training (and the ensuing confidence they have in imparting advice), the limited capacity of the system to cope with large numbers of patients, and the coherence of care provided across the patient's care team.

### Staff Training

#### Limited Knowledge of the Effects of Exercise

Both staff and patients recognized there was a gap in providers' knowledge beyond a certain intensity or duration of exercise. One dietician described how they would impart advice on the basic tenets of carbohydrate counting and the principles of managing exercise but if asked to provide specific information they would instead refer the patient to a consultant.

“The DAFNE and all our in-house carbohydrate counting covers the basic aspects of sport management but we were finding that we were getting people referred for they're going to climb Mount Kilimanjaro, and they're going to… and because it's slightly out of our depth we were finding that we were then referring a lot of those patients up to [specialist consultant] and there was a gap in terms of the basic level of education to the more elite, and whilst I was trying to bridge the gap and give information I didn't really feel fully qualified to give.”

– *HCP2, Diabetes Dietician*

The apparent reluctance of some HCPs to entertain discussions around exercise was also noted by patients. One felt that dieticians were very supportive of the general management of diabetes but hesitant to discuss exercise.

“The dieticians in the formalized setting have been brilliant with everything else, so they would say to me “keep a diary, come back, let's see what did you do here?” And “that's the result of this,” and “you've got the ratios correct, they're working for me”. But the nurses and the dieticians whenever it comes to exercise they step back…”

– *Patient 13*

Some of the carbohydrate counting courses offered to patients incorporated a practical element with patients measuring blood glucose levels before and after a spell of exercise. The HCPs we spoke to raised concerns around their involvement in any such element, at least without appropriate support. For example one Specialist Diabetic Nurse felt unwilling to take patients for a supervised gym session without being accompanied by a specialist member of staff.

“…I wouldn't feel comfortable to take a group of people to a gym for multiple reasons. If it was a trainer now that's different… people who knew what they were doing and specialists in that field then fine, but not if it was just nurse and a dietician.”

- *HCP10, Specialist dietician*

#### Deskilling

The UK's guidance produced by the National Institute for Health and Care Excellence (NICE) recommends that carbohydrate counting courses be delivered by a trained educator (7). One unforeseen consequence of bringing in a specialist educator was that other members of the multidisciplinary team that would otherwise be involved in delivering the programme can become deskilled. One DSN described how the diabetes team were unfamiliar with the format of a new blood glucose diary as they had not been involved in its conception or introduction to patients.

“I think the other thing is what we find when we have got some individual educators for DAFNE or whatever courses that are in a trust, and then what happens is they go back to their regular care, and that the other specialist consultant, or the registrar or the other DSN isn't geared up enough to deal with their new blood glucose diary they're faced with.”

– *HCP12, Diabetes Specialist Nurse*

### Limited Capacity

The number of patients with T1D is growing and staff described how the ability of the service to provide education to all patients was inhibited by limited places on structured education programmes, shortages of relevant staff, and constraints on lengths of consultations.

#### Limited Access to Education Packages

The NICE guidance recommends that all those diagnosed with T1D should be offered a place on a structured education course between 6 and 12 months after diagnosis^1^. However, a consultant in the South West described how places on the course at their hospital were limited and expressed regret that they could not offer a place to everyone eligible.

“It's not for every patient unfortunately. It would be lovely to have it for [everyone]…is just the SWIFT course is completely chocka for the next how many months, because it's multidisciplinary teaching, quite intensive, it takes the days out of their normal work…”

– HCP 7, *Consultant diabetologist*

#### Limited Access to Specialist Providers

The restricted availability of specialist diabetic nurses was reported by one consultant who described how patients were unable to follow a prescribed care plan that involved reviews by specialist nurses because of a lack of appointments within the relevant time frame.

“I will pick up the problem probably so, for those that I don't transfer out, I'll pick up what I think should…set out a plan, and I'll ask the nursing team to pick up in a follow up. The problem we have is actually the nursing clinic appointments are full, they do get longer with patients but there's little availability and sometimes I will have seen the patient again before the nurses have seen them. So that's obviously a barrier to getting good information off.”

– HCP7 *Consultant*

#### Length of Consultation

Both dieticians and consultants described how limits on the time they could spend with individual patients prevented a more detailed dialogue around exercise. The time constraints meant they would prioritize other aspects of T1D management in accordance with patient preference or clinical urgency.

“Time is a nightmare. I think if I go into activity in detail I reckon you're looking at 45 minutes minimum, if you're trying to unpick… so I have a 20 minute slot and I'll spend 45 minutes with someone who's doing regular sport and we're trying to unpick things, because by the time you have unpicked other stuff and you've dealt with other questions …”

– HCP7 *Consultant*

“It's the time element as well; somebody is coming to you with [exercise] just up to a certain level - which may be their weekend football session with their pub team or something - that's okay, and I think that's fine and you can manage that within normal sessions. But if you've got somebody who is becoming a higher level, more competitive, or training for something, and quite big, then it takes a lot of your time up as well and you don't always get *that* amount of time to see the patient.”

– *HCP12, Diabetes Specialist Nurse*

### Coherence of Care

#### Consistency of Message

Within the multi-disciplinary T1D care team staff possess varying levels of training, experience and interest and these differences appeared to lead to patients receiving inconsistent messages from even quite senior staff. For example, one consultant acknowledged that varied levels of awareness of the impact of exercising with T1D might mean advice differs depending on which consultant a patient speaks to.

“So we've got a number of consultants here, and I think you will… there's a couple of us who are much more interested than the others, and I am not sure you would get entirely consistent advice across the consultant group let alone a broader body.”

–* HCP7 Consultant*

Issues emerged around inconsistent information from clinicians with expertise in other specialities whether based in secondary or primary care settings. One DSN described how colleagues in cardiology had experienced a patient with diabetes having hypoglycaemic episodes and so tried to prevent them from attending future cardio-rehabilitation classes.

“We've had people go to the cardiac rehab and we have phone calls because they've had hypos and they want to stop their exercise sessions at the cardiac rehab, and you're like “Yes but we can manage the hypos, the whole point of the cardiac rehab is after their heart attack or whatever else they've had…” and that is then a barrier but it's other healthcare professionals putting that barrier of a person doing the activity.”

– *HCP12, Diabetes Specialist Nurse*

This variation in knowledge of TID between clinicians was also experienced by patients in primary care. One patient described how a locum general practitioner had failed to grasp the differences between Type 1 and Type 2 Diabetes.

“Like the GP thing - when I first started having conversations about the fact that I increase my exercise way back then, and then I was doing DAFNE, we had a locum GP and he said, “Well just low carb anything,” and that was his response, and I went, “I'm Type 1!” he went, “It's the same thing.” So I didn't listen to anything he said…”

– *Patient 2*

#### Continuity of Care

The benefits of continuity of care have been widely recognized across many healthcare settings (34, 35). The knowledge of the physical and mental characteristics of patients gained over time help to inform clinical decision making. In offering individual advice on exercising with T1D staff acknowledged the benefits of building up a relationship with a patient over time, and the increased understanding of how their body responded to exercise.

“It's just really individual. I think though when you are talking to certain people and you might get it right for their training, and then they have a race, and then their training then pushes their blood sugars up so they've got to do something different that day. So it's very hard to go ‘I’ll just go and get from the cupboard, because this is what you need…' because it is really individual, so you have to really know that person, how fit they are, what preparation they can and will do, and then try and tailor it that way to keep them safe and perform well.”

– *HCP2, Diabetes Dietician*

A patient described how continuity of care was inhibited by a perceived lack of communication between primary and secondary clinicians.

“I think part of my problem with my GP surgery is they seem to think all of my care is being done by the hospital, the hospital thinks some of it is being done by the GP and I'm left in limbo with do I need to book an appointment with the GP, do I just wait for my letter to say I'm coming into the hospital?”

–* Patient 8*

### Existing Education Strategies

Current guidance recommends that all patients are offered a place on a structured education package that teaches the basic skills of how to self-manage their condition between 6 and 12 months from diagnosis. The criteria used to determine who should be invited to attend and when was queried by patients. As well as taught courses a variety of written information is also made available to patients via their care provider and the design and content of some of these materials was also questioned.

### Criteria for Invitation to Existing Packages

#### Criteria for Inclusion

Current guidelines recommend a period of 6 months before patients attend a course teaching them the basics of carbohydrate counting and insulin management as this would allow for their T1D to stabilize. However, some patients felt they would have benefitted from attending closer to their diagnosis, feeling the information would have been valuable to them earlier, particularly with relevance to their desire to exercise.

“…I personally feel that if I had been able to access DAFNE when I first started I would have handled it in such a different way. The National Health isn't giving individual choice of at what level you want to consume and take control. So a gentleman who had come on the DAFNE he had just been diagnosed, and it was all just too much for him and basically he had to leave. But for me it was just the most… I wish that I didn't have the complications, if I had DAFNE five years ago I would have been able to handle it and my body would have been a lot better than what it is. So that's one thing that the National Health needs to do, it's one fits all kind of thing.”

– *Patient 13*

Another criterion applied to the selection of patients for attendance on the course was the level of an individual's insulin consumption. One patient reported how they were not invited to attend because staff felt their insulin consumption was too low to justify attendance. However, the patient felt that information contained in the course would still have been useful as they had experienced difficulties in maintaining blood sugar levels from the beginning.

“I think you should be put on it quite quickly, even if you're not necessarily going to use the information they've given you. I wasn't on it until… I didn't go on DAFNE until May, and I think that was too late for me, it was just because I wasn't taking enough insulin to warrant going on it, but I don't think they should really use that, because especially - not necessarily for situations at home, because you know that you're cooking and stuff - but when you go out and things like that? It's really, it's difficult enough at home when you're just starting, it's really difficult to get it right, and I found that I was always way off my insulin when I was out, or if I was on holiday or something like that. But I think even if you're not necessarily going to use the information just have it there so that you can access it if you need to would have been helpful.”

– *Patient 7*

### Format and Content

#### Patient Stories

One educational tool frequently used as part of the structured education package but also via other platforms was the “patient story,” a device that uses real-world examples to provide context for the learning point and so enable patients to more readily assimilate the information^9^. The stories used in T1D frequently involved elite sports stars whose experiences were so different to the patient's experience of living and exercising with T1D that little could be drawn from them^10^.

“Look at the way they performed and they had all the back up in the world that you could ever want. Steve Redgrave is a whopper! His wife is a doctor, so she's there checking him out and pumping him full of insulin when he needs it. Danny McGrain and Mabbutt would have had the sports physios and all the doctors that professional footballers had on tap. Now the rest of us had to do it ourselves.”

– *Patient 11*

In contrast to the stories of famous athletes several patients spoke of the value of the support they gained from people with T1D involved in similar sports and competing at a comparable level.

“I was lucky when I started, I had one of the guys on our mat, higher grade than me, he had been Type 1 since he was six… and I said to him one day, ‘What is happening? I never know if I’m going to be finishing on 3.6 or ten.' He said, ‘that’s the way it is; don't fret because if you fret it will make it go higher.' So that was actually my most useful bit of information I had, ‘don’t fret'…”

– *Patient 9*

“I think it would maybe help more to have somebody that does exercise, somebody that is in this, somebody that's on the same page that lives it and knows it would definitely help.”

– *Patient 1*

#### Generic Written Materials

Educational materials for patients with T1D are provided in a number of formats including booklets and posters. The design and content of these elements was questioned by some of our participants. One patient expressed disappointment at the tone of a poster in a shared waiting area and felt it was uninspiring, particularly during the period they were adjusting to their diagnosis.

“I suppose if you think about the psychology right, you've got all of these hospital appointments that you never used to have to do, you go and sit in some God awful waiting room with all these posters of cartoon characters, about having a carrot or something like that, and it's all this “Here's how your plate's divided.” It's quite patronizing a lot of it, it's just it's not very enticing is it?”

– *Patient 10*

## Discussion

### Key Findings

Though exercise avoidance and physical inactivity are common in patients with T1D this is the first time that the impact of the provision of advice and education on exercising safely has been explored simultaneously with patients and providers. Subsequently we have identified a number of interrelated influences on the success of this provision, these were; the nature of an individual's exercise regime including its intensity, type, and frequency; the degree of patient engagement relating to their preference, the self-management techniques employed, and individual health literacy; Organizational factors that affected staff training, and the capacity and coherence of the system; and finally issues around existing education strategies including access to structured education packages and the content and format of written materials.

### Strengths and Limitations

We have gained a number of novel insights into issues specific to educating patients with T1D on how to exercise regularly and safely. The sample was relatively small and though the intention of this qualitative work was primarily to inform the content and format of a novel education package and not necessarily to reach data saturation, the staff, and patients that took part reflect a varied range of characteristics and by its completion saturation was reached (33). The inclusion criteria for the study meant all the patients we spoke to engaged in regular exercise and included a range of abilities, sports and exercise regimes. We recognize that patients with less ability or interest in exercise might have expressed different views and may need different support and education to encourage them to start to exercise or to exercise more regularly and safely. Similarly it is understood that staff and patients at non-participating sites may have offered alternative opinions due to differences in ethos and experience present at their facility.

### Specific Findings/Existing Literature

#### Staff Training

Deficits in the training of health care providers (HCPs) to deliver advice on exercise were identified. The reported gaps in the knowledge of diabetes specialists reflect broader existing evidence of the repeated failure to equip clinicians with the basic knowledge, confidence and skills to promote physical activity (36, 37). There have been prior calls to train clinicians to prescribe exercise with the same regularity and level of detail as pharmaceutical options (38) and such targeted training has been shown to increase the frequency of exercise counseling for patients with chronic disease (39) including diabetes (40). In the UK and abroad recent initiatives have emerged specifically to educate care providers and patients about exercising with T1D such as JDRF's Performance in Exercise and Knowledge (PEAK) programme (41). However, it seems important that any such training initiative should be offered across the patient's entire care team. This is because we found that not only did dieticians and SDNs describe their lack of confidence in delivering advice on anything but routine and predictable exercise, but patients and staff described the conflicting advice offered to patients between staff groups, specialities, and care settings. One alternative way of ensuring advice remains consistent is to include exercise specialists as part of the existing multi-disciplinary teams (42, 43). However, staff we spoke to reported feeling deskilled as a result of using specialist educators and though effective in the short-term their presence may actually limit the ability of other providers to offer meaningful advice.

#### Lack of Capacity/Adherence to Guidelines

Despite current recommendations exercise did not routinely form part of current consultations among our study participants due at least in part to constraints on time and a reliance on patients to prompt any discussion on exercise. There is a lack of precision in the UK guidelines about the provision of support^1^ for patients wishing to exercise. This is mirrored in the advice of the American Diabetic Association which though making a number of valuable recommendations for patients with T1D regards exercise and carbohydrate intake only suggests that patients may benefit from working with fitness experts that understand diabetes (44). There is no further stipulation of who should educate patients on the principles of managing exercise, when this should be introduced, who should provide continued and responsive support, and the explicit role of self-management (44).

#### Self-Management

In any chronic disease utilizing and improving the capacity of patients to self-manage is a key component of effective care leading to improved patient outcomes, adherence, and efficiency of healthcare utilization (45–48) and this includes patients with T1D (49). The need for effective education to enable patients to self-manage was apparent considering we heard how some keen on exercise failed to understand the importance of self-monitoring blood glucose levels (50). One of the current aims of the UK's National Diabetes Treatment and Care Programme is to improve the uptake of structured education packages designed to “facilitate the knowledge skills and ability for diabetes self-management” (51, 52). Currently the nationally reported take-up is <30% (53) perhaps due in part to the limitations in availability described by some providers we spoke to.

#### Existing Provision

The lack of capacity within the system places more importance on sources of educational material that can be independently accessed. The design and content of educational material for patients deserves careful consideration as it influences their satisfaction, adherence, and health outcomes (54) yet patients' needs are varied and their ability to independently assimilate this information determined by numerous factors including age, socio-economic status, education, and ethnicity (55–58). Some we spoke to described their antipathy toward aspects of existing diabetes educational material, including generic “diabetes” posters that failed to differentiate between T1D and Type II Diabetes. One particular source of frustration amongst patient participants was selecting elite sportspeople to tell their patient story^11^. Used appropriately these stories can be a powerful tool and through their familiarity, become especially vivid (35) yet many existing examples bore little resemblance to patients' everyday experience of living and exercising with T1D. Instead patients spoke of the value of peer-to-peer contact where the benefits of discussing relatable strategies and solutions would be more readily applicable. The benefits of this type of peer-to-peer mentoring in diabetes have been recognized previously and appear an underutilized resource in supporting those wishing to exercise (59).

## Conclusion

Drawing on both patient and staff perspectives, our findings provide persuasive evidence of the need for a more responsive and directed education programme for patients with T1D that exercise regularly alongside a similar programme for providers to enable them to offer ongoing advice for this level of activity. Currently there is no such educational package available for patients with T1D and the findings we present here will inform the content and delivery of an educational package that can support patients not only in the UK but also internationally. The nascent EXTOD education package will be offered to all patients based on their interests and preferences and not dictated solely by the time since diagnosis. It will contain advice on the management of regular and irregular bouts of aerobic and anaerobic exercise alongside how to prepare for specific events. The patient stories utilized will more closely reflect the everyday experiences of patients with T1D supported by group education that both facilitates peer-to-peer interaction and minimizes costs to the provider. Perhaps most importantly it will incorporate a section that trains staff across the diabetes care team to enable them to contribute to the course directly or more confidently offer applicable advice as part of their routine patient contact.

## Author Contributions

The overall concept of the project was devised by RA and PN. The data collection was conducted by IL and the analysis performed by IL and SG. IL produced the original draft this was then critically revised by SG, RA, and PN for important intellectual content. A further draft was produced and all authors approved the final submitted manuscript. Each author certifies that this material or similar material has not been and will not be submitted to or published in any other publication. IL is the guarantor who takes full responsibility for the work as a whole, including, access to data, and the decision to submit and publish the manuscript.

## EXTOD Education Programme Development Team

Yvonne Doherty, June Sorensen, Janette Barnette, Ian Gallen, Dinesh Nagi, Melanie Davies, Lindsey Apps, Janet Gorton, Heather Daley, Catherine Thompson, Manyee Li, Barbara Hudson.

### Conflict of Interest Statement

The authors declare that the research was conducted in the absence of any commercial or financial relationships that could be construed as a potential conflict of interest.

## Footnotes

^1^https://www.nice.org.uk/guidance/ng17 (accessed October 2017)

^2^American Diabetes Association Diabetes Care (2018). 41(Supplement 1):S38–50.

^3^http://www.isrctn.com/ISRCTN61403534?q=&filters=&sort=&offset=5&totalResults=14839&page=1&pageSize=10&searchType=basic-search(accessed November 2017)

^4^https://www.bertieonline.org.uk/ (accessed November 2017)

^5^https://www.nhs.uk/conditions/type-1-diabetes/living-with/ (accessed October 2017)

^6^https://www.kingsfund.org.uk/projects/gp-inquiry/patient-engagement-involvement (accessed October 2017)

^7^https://www.england.nhs.uk/ourwork/patient-participation/self-care/ (accessed November 2017)

^8^https://www.hee.nhs.uk/our-work/health-literacy (accessed November 2017)

^9^https://improvement.nhs.uk/documents/2140/patient-stories.pdf(accessed November 2017)

^10^https://www.diabetes.co.uk/celebrities/gary-mabbutt.html(accessed October 2017)

^11^http://www.diabetesadvice.co.uk/real-stories/sir-steve-redgraves-winning-ways-with-type-2-diabetes (accessed October 2017)
