# Supplementary material for: Patient and Healthcare Professionals Perspectives on the Delivery of Exercise Education for Patients With Type 1 Diabetes
Source: Front Endocrinol (Lausanne). 2019 Feb 19;10:76. doi: 10.3389/fendo.2019.00076 (PMC6390874; doi:10.3389/fendo.2019.00076)
Supplement: Supplementary file 1 [file Table_1.DOCX]

| **Theme** | Sub theme 1 | *Patient* | *Staff* |
| --- | --- | --- | --- |
| **1.0 Exercise regime** | 1.1 Type of exercise | *Predictable intensity*  Truly does anybody really know what they’re going to do, unless they’ve got a personal trainer saying what they’re going to be doing for how many minutes?” P9  Definitely with the intensity, because that vary depending on just even how you’re feeling on the day regardless of levels, you might not feel like you want to go too far one stage and therefore you get different readings than what you might have done a week ago doing the same distance, or length in time of exercise or whatever. But the intensity I think has a big impact. P8 | *Predictable intensity*  I had somebody that was doing triathlon and things, and we had got it sorted out and it was going really well until he had his first race, and his blood was 20 that morning because of all the excitement of it all. But he couldn’t leave it, but in the end gave just about a unit and that was enough to bring him down so he didn’t get a hypo. But you forget about that when you’re busy just concentrating on all the individual sports. But when you’re then competing that’s actually going to be different to all the training you’ve done, and that’s hard. HCP5  *Anaerobic sport*  I still find it a bit hard around the anaerobic sport, when they’re doing weights and things like that, and actually their blood sugars go up, so one guy I mentioned he does that first thing in the morning, so he has no breakfast and three units of insulin and goes to the gym, and then his blood sugars are okay. I sort in my head think oh [laughter] but it does work. But that took a lot on both of us gradually edging that up, but it doesn’t seem right does it? HCP5  *Specialised sport*  I think my difficulty comes when I’ve got people who are very sporty, either competitive sport or are training for something specific like for a marathon or something. Then I feel it stops my confidence not theirs, and that might rub off on them, I don’t know, because then it becomes quite difficult, because you’re managing quite a lot regarding their diabetes, and I suppose that’s just experience of what to do with people that are very sporty or training. HCP10 |
|  | 1.2 Patterns of exercise | *…* | *Routine vs sporadic*  Obviously when we’re talking about say aiming for 20 to 30 minutes a day I think they think that has to be a run round the block, or gym, … because there’s only generally a handful of people that do a sporting or regular activity, and sometimes that separates the group a little bit because there’s some who sit at the back who don’t do any of that…those elite ones want the more advanced information, those that are just doing the general day to day activity probably would be confused by all this extra information… HCP4 |
|  | 1.3 Intensity | I used to event, luckily I don’t event anymore, I just do dressage, and then dressage horses so it’s very controlled, so it’s not a huge high impact thing. You work hard but your heart rate never… it probably does through adrenaline, but not like a run or if you’re cycling hundreds of miles, it’s quite controlled. But I find that… I find it difficult to eat anything sweet because it puts my blood up too quickly, I get headachy and I can’t concentrate very well. So I find it easier like you were saying to eat well the night before and then I have some cereal for breakfast whatever, normal insulin ratios, and then throughout the day I would eat things like bananas or Alpen bars, or Nutrigrain bars, and I find that’s easier, and I can maintain myself all day like that, whether it’s right or wrong, it’s just what’s worked for me. P6 | I think some of the patients I have seen that are fairly elite seem to have the poorer control, their control isn’t great, and I think it’s because it’s so hard to manage, because if you think they’re setting targets of what their blood sugar should be before they exercise, that’s higher than maybe some of the targets pre-meals, so they’re always constantly running higher, and then when you’ve got the competitive nature then they’re obviously high, and suddenly adrenaline based sports it’s so hard to keep combating HCP4 |

| **2.0 Patient engagement** | 2.1 Patient preference | *Patients*  *Priority of exercise*  So I was proactive a few years ago and okay well I don’t think my food stuff is working properly, and that’s when I started doing the carb loading the day before, doing the stuff. So I emailed in and said I would like to talk over this with somebody to make sure I’m not doing any damage, and they made me an appointment with the dietician, and I had it six weeks later. So you’ve just got to ask. P2 | *Staff*  *Priority of exercise*  It’s a question that you will ask, and sometimes then you know it needs to be a little bit more around that, and some people would say don’t really exercise. So it’s individual really. HCP9  I think I usually respond to patient requests rather than being proactive. HCP6 |
| --- | --- | --- | --- |
|  | 2.2 Self management | *Monitoring*  The same with that as well, and it took me ages to work it out as well, and I found that if I did eat a really good pasta dinner the evening before and I knew I was competing the next day actually I kept my background insulin the same, I never change that whatever I’m doing, but I might have to have a lot less insulin, in fact sometimes I have no insulin when I’m competing, and then it can be difficult because adrenaline you can get a real false reading, and I can be driving, because I’ve got an HGV licence as well so I have to be really careful how quick it comes down. So I can leave a competition and it can be nine or something, or ten, and then I can drive an hour and a half home and it can be five, it’s that kind of… and I tend to have a four hour window after exercising of how much insulin, I have to halve my insulin four hours after I’ve finished competed, so have to juggle it around quite a lot, trial and error. P6  *Carbohydrate counting*  Mine is a little bit involved with diet as well. So I tend to reduce the amount of insulin I’m having if I’m training, but if I’m performing at the weekend I tend to carb load the day before, and then I have less but fully protein based the day off so that my liver doesn’t going into overdrive with my blood sugar spikes. It took me quite a while to work out how to manage that. P2 | *Monitoring*  *…*  *Carbohydrate counting*  So anyone who I’m giving exercise advice to I find it very difficult to do anything if they’re not carb counting. We I find it very difficult if they’re not carb counting, we do it when they’re not carb counting, but we will always discuss carb counting specifically if they haven’t already done it, and by no means all of them have done, we’ve actually found quite a lot of people aren’t as interested as you might think. HCP12 |
|  | 2.3 Health Literacy | *Understanding advice*  But the exercise is a whole grey area, I just can’t… like you’re saying about knocking off a couple of units, then I’m riding on a high, and I think okay let me just knock off four, and then I get a hypo. Exercise is a minefield for me, and it’s really sad, because it scares me often, I have to carry stuff with me, which you need to anyway, but there doesn’t seem to be a formula. P13  I’ve found actually that the things like the booklets are more helpful really because you haven’t always got access to the internet when you’re in the middle of a session, and you think hang on a minute, and you just get your book out of your bag and there it is, hard copies of things might be better. P3 | *Understanding advice*  Talk about the talking test, whether they can talk and how red in the face they go, that’s what we talk about, to calibrate it. HCP7  There’s another quite challenging cohort who use the sport in order to manage their diabetes rather than just doing it because they want to do it. So you do get people who wake up with a high blood glucose and that day they will get on their exercise bike and really go for it, because in order to try and… so they’re quite an interesting group really. I think that happens quite a lot. HCP3 |

| **3.0 Organisational factors** | 3.1 Staff training | *Patients*  *Limited knowledge of the effects of exercise*  It’s all the way across because I was called in only last week actually with the GP, and she said to me, “What are you doing with the testing strips, are you eating them?” Those were her words, and I said, “What do you mean?” And she said that, “Well the way we’ve calculated you should be testing twice a day…The National Health Service has spent a lot of money sending me to DAFNE and as a result I don’t do it for love, but I have to do this. So even the GPs you have to… P13  I was told have a banana after your class, that was what I was told, because I said I liked bananas, “No problem, just have one after your class…[that was the] consultant .” P9 | *Staff*  *Limited knowledge of the effects of exercise*  I had a lady yesterday who swims 40 lengths three times a week, and had a massive hypo in the pool a week ago, had to be dragged out, and she is not ever going near water again. So these sort of things are also part of their experience, it’s going to have a big impact, and then you need to be able to come in and say… and that’s where I do struggle as well about the right carbohydrates, the best isotonic drink and all that sort of thing, my knowledge of that is limited. So I think I didn’t feel I could really help her in managing this, and that’s potentially going to be an enormous issue for her. HCP4 |
| --- | --- | --- | --- |
|  | 3.2 Capacity | *…* | *Limited access to education packages*  it’s not for every patient unfortunately. It would be lovely to have it for… because again time, I am going to bring it up, is just the SWIFT course is completely chocka for the next how many months, because it’s a multidisciplinary teaching, quite intensive, it takes the days out of their normal work, and it’s really. Everybody has got fantastic things to say. I wish it was for everybody where it’s not for everyone, usually it’s clinicians who refer, or the diabetic nurses picks up patients who need to go onto the SWIFT course. That’s one of the reasons we started this three monthly unofficial just teaching kind of thing, which actually gives more support to the newly diagnosed. HCP13  Also with the more people you did [through the education package] the more people you had to invite [to a refresher course], and then you didn’t know how many were going to come, and a big hall or room this size… It was just too complicated to organise. HCP5 |
|  | 3.3 Coherence of care | *Coherence of care*  P6: The guy you see he’s a specialist here, he’s a specialist exercise guy or…? P1: …well he’s into his sport anyway and he’s… P6: I had no idea. P1: Yes. P6: I had absolutely no idea and I’d love to have seen him, love to have. | *Consistency of message*  We’ve got it [specialised knowledge] across those of us who would deal specifically with pumps and who do the problematic Type 1s but it’s not quite the whole team as yet. HCP12 |

| **4.0 Existing education strategies** | 4.1 Structured education package | *Patients*  *Criteria for inclusion*  I think something both of you have touched on, what you said about the national health and what you said about… I personally feel that if I had been able to access DAFNE when I first started I would have handled it in such a different way. The national health isn’t giving individual choice of at what level you want to consume and take control. So a gentleman who had come on the DAFNE he had just been diagnosed, and it was all just too much for him and basically he had to leave. But for me it was just the most… I wish that I didn’t have the complications, if I had DAFNE five years ago I would have been able to handle it and my body would have been a lot better than what it is. So that’s one thing that the national health needs to do, it’s one fits all kind of thing. P13  Generally they say you have to wait a year before you go on a DAFNE course, which is bonkers. I think you should be put on it straight away. P7  I think you should be put on it quite quickly, even if you’re not necessarily going to use the information they’ve given you. I wasn’t on it until… I didn’t go on DAFNE until May, and I think that was too late for me, it was just because I wasn’t taking enough insulin to warrant going on it, but I don’t think they should really use that, because especially not necessarily for situations at home, because you know that you’re cooking and stuff, but when you go out and things like that it’s really, it’s difficult enough at home when you’re just starting, it’s really difficult to get it right, and I found that I was always way off my insulin when I was out, or if I was on holiday or something like that. But I think even if you’re not necessarily going to use the information just have it there so that you can access it if you need to would have been helpful. P7 | *Staff*  *….* |
| --- | --- | --- | --- |
|  | 4.2 Format and content | *Patient stories*  Yes, and I think you need patient advocates who can go into a ward if someone wants to talk to someone who has diabetes, because I didn’t know anyone who had diabetes, I still don’t apart from when I go to things like this, I’m the only diabetic that I know in my day to day like…. I was for 35 years, it’s only last year when I came to the DAFNE course I met other type 1 diabetics. P10  *Generic written information*  P9: Widen the images of exercise, and please don’t do as you say the smiley face cartoon mouse approach. Moderator: A little bit of reality perhaps? P9: Please, yes P12: Picture of a big strong man with a needle in his arm or something like that  If you think about the messaging people get about diabetes it’s either someone grinning from ear to ear on roller-skates, or it’s someone with an amputated leg [laughter] there is a middle ground, you could show normal people doing normal things. It’s like just do these things and then you can live a normal life, no big deal, but you’ve got to do all this extra admin to get to that point. You’ve got a deficiency that normal people don’t have, but it’s either one thing or another, it’s either you’re a big fat blob your leg is going to get chopped off, or look at Steve Redgrave he got gold, well good for him quite frankly. So that’s the… there’s confusion in the NHS, they don’t want to be too nanny state, and at the same time they’re not being realistic with people about what they need to do is my take on it. P10 | *…* |
